# The role of human capital and stress for cost awareness in the healthcare system: a survey among German hospital physicians

**DOI:** 10.1186/s12913-024-10748-z

**Published:** 2024-03-07

**Authors:** Christoph Lüdemann, Maike Gerken, Marcel Hülsbeck

**Affiliations:** 1 Witten/Herdecke University, Alfred-Herrhausen-Str. 50, 58455 Witten, Germany; 2 University of Applied Sciences Munich, Hohenzollernstr. 102, 80796 Munich, Germany

**Keywords:** Health economics, Health expenditure, Cost awareness, Cost estimation, Human capital, diagnosis related groups

## Abstract

**Background:**

Germany has the highest per capita health care spending among EU member states, but its hospitals face pressure to generate profits independently due to the government’s withdrawal of investment cost coverage. The diagnosis related groups (DRG) payment system was implemented to address the cost issue, challenging hospital physicians to provide services within predefined prices and an economic target corridor to reduce costs. This study examines the extent of cost awareness among medical personnel in German hospitals and its influencing factors.

**Methods:**

We developed an online survey in which participants across all specialties in hospitals estimated the prices in euros of four common interventions and answered questions about their human capital and perceived stress on the workplace. As a measure of cost awareness, we used the probability of estimating the prices correctly within a reasonable margin. We employed logit logistic regression estimators to identify influencing factors in a sample of 86 participants.

**Results:**

The results revealed that most of the respondents were unaware of the costs of common interventions. General human capital, acquired through prior education, and job-specific human capital had no influence on cost awareness, whereas domain-specific human capital, that is, gaining economic knowledge based on self-interest, had a positive nonlinear effect on cost awareness. Furthermore, an increased stress level negatively influenced cost awareness.

**Conclusions:**

This paper is the first of its kind for the German health care sector that contributes responses to the question whether health care professionals in German hospitals have cost awareness and if not, what reasons lie behind this lack of knowledge. Our findings show that the cost awareness desired by the introduction of the DRG system has yet to be achieved by medical personnel.

**Supplementary Information:**

The online version contains supplementary material available at 10.1186/s12913-024-10748-z.

## Introduction

As clinicians substantially influence about 35% of a hospital’s total expenses [1], there is a concern that medical personnel in (German) hospitals may prioritize profit considerations when making decisions [2]. Previous studies in European countries have already addressed this issue and found that apparent cost-driven decisions by medical personnel are countered by the lack of knowledge for economic decision making as they do not know the costs of medical supplies or interventions [3–6]. Therefore, cost-driven decisions might be based on a hunch at best [7–9]. It is still unknown what kind of knowledge leverages the cost awareness of medical personnel and which additional organizational factors influence this. Drawing on the concepts of general vs. specific human capital, this paper is the first to investigate distinct kinds of human capital as drivers of cost awareness. Moreover, we argue that the quality of working conditions, such as work-related stress, will negatively impact this cost awareness [10–12]. Cost awareness is commonly defined as someone’s knowledge of costs [13, 14]. As early as 1994 a study showed that cost awareness among physicians is a valid measurement of physician attitudes and their influence on resource utilization [15]. Ryskina et al. (2015) figured that the training environment may play a role in shaping physician cost awareness later in their careers [16]. Job-related stress was already described as a relevant factor by Dyrbye et al. (2019), who found that burnout among medical staff is not only associated with self-reported unprofessional behaviors but also less favorable cost-awareness [17]. The purpose of this study is to empirically investigate different forms of human capital and job-related stress as drivers of the cost awareness of common medical interventions among medical personnel working in all specialties in hospitals. Our basic hypothesis was that higher human capital leads to higher cost-consciousness. Additional hypotheses that arise from this are the questions of whether general human capital or specific human capital led to better estimates.

## Background

Per capita spending on health care is higher in Germany than in all other member states of the European Union. In 2019, 4,505 euros were spent on patient care in Germany– 28% more than the EU average. Countries with comparable life expectancy spend less [18]. German hospitals are under pressure to generate profits from their daily business, since the agreement for states to cover investment costs is no longer being upheld [19]. At the same time, the reasons for the above-average costs in the German healthcare system are manifold. Fragmentation of the system and the insufficient coordination of patient treatment play a leading role. Also, the transfer of specialist care to hospitals is a major cost driver for the overall system as well [20]. In Germany, hospitals account for the largest share of healthcare expenditure by statutory health insurers, at more than 34% [21, 22].

German hospitals are confronted with the necessity to weave in revenues from the diagnosis related groups (DRG) payment system into their day-to-day operations. The introduction of the DRG system was linked to the goal of creating more transparency, both on the service and cost side. It substituted the self-cost recovery principle in which patients or respective payers are billed for the hospital services on a flat-rate basis using the hospital’s own costs estimated from a previous period [23]. The DRG system itself was thought to be a solution for the cost problem and was aimed at hospital physicians that were now faced with the challenge of providing their services at predefined prices and thus within an economic target corridor. Before the introduction of the DRG system, hospitals were required to submit a service and cost statement to the health insurance funds for upcoming budget negotiations, which also provided information on the cost structures of the respective hospitals [23]. According to the principle of self-cost recovery, any profits made by hospitals at that time remained with the health insurance funds. The switch to the DRG system should now ensure that profits, on the other hand, can remain with the hospitals. The obligation to disclose cost structures also ceased to apply with the introduction of DRGs. The objective to generate profits remains crucial, particularly given the high and continually rising costs in the hospital sector.

The abolition of the principle of self-cost recovery created an asymmetry of information that now runs through the entire organization, right up to the patient’s bedside. There, the economic pressure that arose from the systematic failures of German health care system, hospital workers are blamed for their part in the seeming economic conspiracy toward the patients. Manzei et al. describe numerous empirical studies that show the serious negative effects of economization and the associated new paradigms on the patients’ experience of illness, the work organization of nurses and physicians, and economic efficiency [24–28]. If, for the sake of cost efficiency, primarily personnel costs are saved, e.g., fewer nursing staff are available per patient, the demand for economic efficiency meets the impossibility of providing the services of a hospital. Within the hospital, personnel and material costs come into focus. Regarding the material costs, physicians and nurses are among those who have a significant influence on the use of medical resources through the indication and their daily work [29] and are now forced to pay attention to costs. The logic of cost-driven medical decisions competes with the logic of helping the patient.

Meanwhile, the lack of cost awareness is a widespread phenomenon among almost all players involved in healthcare [30]. The effects that increased cost awareness can have in the healthcare system was investigated, among others, by Monsen et al. [31]. Sorber et al. (2020) performed a cross-sectional study of surgeons, trainees, nurses, and surgical technicians across all surgical specialties. It was found that price of common supplies was globally impaired for the entire surgical team [7]. Glennie et al. (2019) investigated whether improving the transparency of material costs incurred during a surgical intervention can reduce the overall cost of the procedure. Costs associated with spine surgery interventions were documented during a five-month period in which surgeons did not have access to cost information. In the following five months, the costs were documented again. During this period, cost information was available to operating physicians. There was a significant cost reduction in the period with improved cost awareness. Furthermore, with pronounced cost awareness, total costs of the procedures could be significantly reduced [8]. A study by Ryan et al. used a survey format to examine cost awareness within surgical hospitals in Ireland [3]. Only 14% of the respondents were close to the actual costs with their estimates. The work experience of the respondents had no influence on the size of the discrepancy between the estimate and actual costs. Similar results were shown in studies by Hernu et al. (2015) [9] and Kynaston et al. (2017) [13]. In a detailed study, Simon et al. observed that, under the impression of budget caps and the introduction of flat rates per case and special charges, decisions about admission, care, and transfer or discharge of patients are increasingly permeated by economic calculations [30]. Marckmann et al. (2011 and 2014) addressed the question of how physicians can incorporate cost considerations into their microlevel decisions in a medically rational and ethical manner [32, 33]. These examples indicate that cost awareness in hospitals and cost awareness among medical personnel have the potential for an overall cost reduction within a healthcare system. The question addressed is whether health care professionals in German hospitals have cost awareness and if not, what reasons lie behind this lack of knowledge.

## Method

### Survey overview and data collection

To answer our research question and investigate the drivers of cost awareness among physicians in German hospitals, we developed an online survey specifically for this study based on previous research [3, 14, 34]. The survey contained four main parts. In the first part, the participants estimated the prices in Euro of four common interventions: colonoscopy, cholecystectomy, coronary stent, and pacemaker. We developed the estimation questions based on previous studies and defined the price of the interventions as returns meaning the sum of the material and personnel costs required for cost unit accounting, according to the manual for the calculation of treatment costs of the *GKV Spitzenverband*, rather than the whole complexity of a patients stay that would be taken into account in a lump sum per case [3, 14, 34]. The answers in the remaining parts of the survey were made on a 6-point Likert scale. In part two, participants were asked to indicate their level of different kinds of human capital, that is, their level of economic knowledge received through formal education (*general human capital*), their level of knowledge in the economic domains of controlling, marketing, insurance, tax, human resources (*domain-specific human capital*), and their level of economic knowledge accumulated through job tenure (*job-specific human capital*). To control for a potential self-selection bias, that is, that participants took part in the survey because they are interested in economics, we asked in how far participants trained themselves on economic topics in their spare time (*own training in economics*) and in how far they think economically when they use material at work (*think economically when using material*). The third part inquired about working conditions asking about the general level of *stress* on the job using an existing scale [35], and the extent to which participants can work free of *cost-related stress*, that is, stress caused by the fact that participants are under economic pressure and have to pay attention to costs at work. In the last part, we controlled for demographic data: *age, gender*, *job role* (physician or nurse), and whether participants had a *supervisory position*. The survey was pre-tested on five members of the target-population to gain feedback regarding the comprehensibility of the questions. No changes were made. The final survey took about six minutes to answer. An overview of the survey questions can be found in the supplement.

Data collection took place in May and June 2021 in hospitals in Germany. We distributed our online survey among clinicians in German hospitals through e-mail and social media channels. The target group consisted of clinicians, i.e., registered nurses and physicians with direct patient work. We strived for diversity in terms of size of the hospital, form (academic and regional hospitals) and departments of the respondents in the sample ensuring a representative study cohort. The authors obtained permission to survey staff in personal discussions with the respective heads of the medical and nursing personnel. Furthermore, the invitation included an explanation of the background of the survey, informed consent, the approximate length of the survey, and a reference to the General Data Protection Regulation in Europe. Participation was anonymous, and participants were identified only by their IP addresses to eliminate potential double participation. By filling out the survey, the participants agreed to the processing of the anonymized data. We sent a reminder two weeks after the initial distribution of the survey. Taking part in the survey was voluntary and participants did not receive compensation for their participation.

In total, 137 participants accessed the survey. We removed double entries based on IP addresses and incomplete surveys. The final sample consisted of 86 participants. About 50% of the respondents were nurses and 47.5% of the respondents were physicians. Another 3% of the respondents were from the professional group of the so-called support staff. About half of the respondents were women (54%), and about 28% had a supervisory position. On average, respondents were 38.5 years old (SD = 9.75), and they worked in their job for 14.25 years on average (SD = 10.5).

### Data analysis

We transferred the data to Stata 16 for statistical analysis. We followed established protocols for the analysis [3]: first, we calculated the distance to the correct estimation for each intervention using absolute values. As we were interested in the overall cost awareness of clinical personnel, we aggregated the four interventions, resulting in 344 ratings (each participant rated 4 interventions, resulting in 344 ratings from 86 participants). Second, we calculated what percentage of the prices were estimated correctly overall in general. Following previous research [3], we considered the ratings that were in the 25th percentile above and below the correct price to be valid because an exact estimate is not a realistic assumption. Third, we set up a dummy variable, which took the value one for the correct estimate of the price and zero for an incorrect estimate of the price. We predicted the correct estimate using logistic regression analysis and clustered standard errors. We used clustered standard errors because the prices for each estimated intervention were filled out by the same respondents and thus ratings might be correlated. The findings showed that only about 18% of the interventions were correctly estimated. To ensure the robustness of the findings and avoid potential measurement bias, we therefore confirmed the correct estimates using probit regression and rare event logistic regression analysis. Probit regression is more robust to potential influential outliers, whereas rare event logistic regression was developed specifically for datasets where the outcome of interest is rare, thus taking into account that only 18% of interventions were correctly estimated.

## Results

### Descriptive statistics

Table 1 shows the descriptive statistics. The statistics for the four interventions show high standard deviations, indicating a high variance in the estimations. This may be a first hint towards the fact that respondents lack economic knowledge to correctly estimate the interventions. Respondents rated their domain-specific human capital and their general human capital (taught during their studies) as generally low but indicated that they often think economically when using material and that they trained themselves in economics. However, they rated their cost-related stress to be moderate, while they reported their job-stress level as high.

**Table 1:** Descriptive statistics

Variable	Mean	Std. dev.	Min	Max
Colonoscopy	776.49	934.62	100	5000
Cholecystectomy	3235.55	3734.27	100	25,000
Coronary stent	2742.30	3316.18	150	20,000
Pacemaker	3178.52	3868.15	100	20,000
General human capital	2.96	1.51	1	6
Job-specific human capital	14.25	10.58	1	42
Domain-specific human capital^a^	2.92	1.16	1	6
Own training in economics	3.14	1.67	1	6
Thinking economically when using material	4.56	1.23	1	6
Job-Stress^a^	4.24	1.16	1	6
Cost-related stress	3.07	1.52	1	6
Gender	0.54	0.49	0	1
Physician	0.54	0.51	0	1
Supervisory position	0.28	0.45	0	1

*Note N* = 86; ^a^Cronbach’s α. the factors domain-specific human capital and job-stress consist of five items each. Therefore, we assessed the internal consistency. Domain-specific human capital α = 0.88; Job-Stress α = 0.80

### Estimation of correct prices

Our goal was to examine which factors significantly affect the correct estimation of the prices. Table 2 shows the results of the regression analysis based on 344 ratings. General human capital and job-specific human capital had no influence on price estimation. Domain-specific human capital had a negative and significant effect on the estimation. Because domain-specific human capital can have a non-linear effect, we included the square of the variable to test this assumption. Domain-specific human capital can accumulate through learning and experience, and the rate of learning may vary over time. This can lead to non-linear effects where individuals may experience steeper learning curves early in their careers, which then might lessen as they gain more experience. The results show that higher domain-specific human capital had a significantly negative influence on the estimation. Next, we found indication that participants, who thought economically about their use of materials, were better at estimating the price. Finally, the level of stress had a significant negative influence on the estimation. The results of the robustness checks, depicted in the appendix, confirmed the findings.

**Table 2:** Results of Logistic Regression with Price Estimation as Dependent Variable

General human capital	-0,10	
	(0,09)	
Job-Specific human capital	-0,00	
	(0,01)	
Domain-specific human capital	**-0,56**	***
	**(0,20)**	
Domain-specific human capital squared	**0,12**	***
	**(0,04)**	
Own training in economics	-0,06	
	(0,09)	
Thinking economically when using material	**0,11**	*
	**(0,06)**	
Stress	**-0,23**	***
	**(0,08)**	
Cost-related stress	0,03	
	(0,11)	
Gender	-0,46	
	(0,37)	
Physician	-0,12	
	(0,42)	
Supervisory position	-0,36	
	(0,35)	
constant	0,05	
	(0,54)	

*Note* *p > 0.10, **p > 0.05; ***p > 0.01; Data analyzed based on 344 ratings

## Discussion

The findings of this exploratory study suggest that neither general human capital acquired through prior education nor job-specific human capital acquired through work experience had an impact on accurately estimating prices. Although these results add to earlier studies such as Ryan et al. (2018), [3, 7, 9] which reached similar conclusions in other countries than Germany, it is surprising that job-specific human capital had no influence. One would expect that with increasing work experience and thus prolonged exposure to decision making regarding the costs, cost awareness would also increase. Therefore, the possession of general and job-specific human capital seems not sufficient to make informed cost-related decisions and allocate resources effectively. More interestingly, we found a nonlinear effect of domain-specific on accurate cost estimation, suggesting that possessing expertise in specific economic domains beyond a certain threshold improves cost awareness and thereby decision-making. While the probability of accurate price estimation diminishes with higher domain-specific human capital at first (linear effect), it increases after reaching a certain threshold. It is questionable whether the initial negative effect is due to overconfidence in the less-skilled [36, 37] or points to the dangers of hunches [7–9]. At the same time it illustrates the need to leverage the existing domain-specific human capital through improving metacognition and reflection in clinical settings [38] to cross the aforementioned threshold. Moreover, the nonlinear effect clearly points to the fact that investments into domain-specific human capital only pay off after a certain inflection point. A certain level of knowledge is necessary to make precise cost estimations. Failing to meet this threshold could result in inaccurate estimations, emphasizing the risks associated with having an incomplete understanding of economics. Furthermore, adopting an economic mindset when using material was found to have a positive and significant influence on price estimation, pointing to and controlling for a certain self-selection of economically minded individuals into the sample. Finally, and unsurprisingly, unfavorable working conditions, estimated by job-related stress, had a negative influence on estimating the correct prices.

The limitations of this study offer starting points for further research. Only 18% of the costs were estimated correctly. This could be due to a possible self-selection bias, as only clinicians interested in the topic participated in the study. However, the rather low number of correct estimates has also been found in previous studies [3, 14]. Nevertheless, future studies should be conducted with a larger sample. Next, participants assessed their economic knowledge themselves, which can lead to a possible over- or underestimation of their knowledge. Future research could complement the self-assessment by external information from peers. Looking at the cost awareness in isolation, without being able to meaningfully contextualize the quality of care, is a challenge. Meanwhile, the quality of care is hard to objectify. Nevertheless, the results showed that cost awareness plays a critical role in the German healthcare system. Therefore, future research could examine the relation between cost awareness and the quality of care by analyzing secondary data from hospitals. First studies from other countries [29, 39] hint at the notion that increased cost awareness led to a reduction of operation costs without reducing the quality of care.

## Conclusion

Overall, the findings of this study suggest that clinicians in German hospitals may have incomplete knowledge and must rely solely on self-interest to gain economic knowledge. This has practical implications, including the need to repeatedly address cost awareness on the job. For example, clinicians could examine costs at meetings after operations to break down the costs per patient, and quality management or financial departments could provide specific ward costs. Additionally, these results imply that current medical and nursing curricula should include more economic and health economic topics. Uthoff et al. (2019) [40] already indicated in their study the necessary integration of economic subjects in medical curricula to help physicians manage ethical conflicts between economics and medicine for the benefit of patient care. Thus, formal education could promote interest and provide medical personnel with a basis to make informed decisions.

### Electronic supplementary material

Below is the link to the electronic supplementary material.

Supplementary Material 1

Supplementary Material 2

Supplementary Material 3

## Data Availability

The dataset used and analyzed during the current study are available from the corresponding author on reasonable request.

## Abbreviations

DRG: Diagnosis related groups
