# Supplementary material for: The role of human capital and stress for cost awareness in the healthcare system: a survey among German hospital physicians
Source: BMC Health Serv Res. 2024 Mar 7;24:310. doi: 10.1186/s12913-024-10748-z (PMC10921634; doi:10.1186/s12913-024-10748-z)
Supplement: Supplementary file 1 — Supplementary Material 1 [file 12913_2024_10748_MOESM1_ESM.docx]

**Supplementary Information**

| **Table S1. Correlations** |  |  |  |  |  |  |  |  |  |  |  |  |  |  |
| --- | --- | --- | --- | --- | --- | --- | --- | --- | --- | --- | --- | --- | --- | --- |
| Colonoscopy |  | (1) | (2) | (3) | (4) | (5) | (6) | (7) | (8) | (9) | (10) | (11) | (12) | (13) |
| Cholecystectomy | (2) | -0,41* |  |  |  |  |  |  |  |  |  |  |  |  |
| Coronary stent | (3) | -0,15 | 0,40* |  |  |  |  |  |  |  |  |  |  |  |
| Pacemaker | (4) | -0,01 | 0,20* | 0,56* |  |  |  |  |  |  |  |  |  |  |
| General human capital | (5) | 0,01 | 0,10 | -0,02 | -0,13 |  |  |  |  |  |  |  |  |  |
| Specific human capital | (6) | -0,10 | -0,02 | 0,15 | -0,02 | -0,19* |  |  |  |  |  |  |  |  |
| Domain-specific human capital | (7) | 0,10 | -0,16 | -0,01 | -0,02 | 0,31* | 0,25* |  |  |  |  |  |  |  |
| Own training in economics | (8) | 0,11 | -0,09 | 0,03 | 0,12 | 0,19* | 0,24* | 0,64* |  |  |  |  |  |  |
| Thinking economically when using of materials | (9) | -0,02 | -0,02 | 0,11 | 0,01 | 0,03 | 0,32* | 0,26* | 0,35* |  |  |  |  |  |
| Stress | (10) | 0,09 | 0,16 | 0,06 | 0,14 | 0,07 | -0,22* | 0,04 | 0,15 | 0,04 |  |  |  |  |
| Cost-related stress | (11) | 0,06 | -0,23* | -0,27* | -0,21* | 0,07 | -0,05 | -0,15 | 0,01 | -0,16 | -0,35* |  |  |  |
| Gender | (12) | 0,16 | 0,05 | -0,01 | -0,07 | 0,07 | 0,12 | -0,01 | -0,14 | 0,08 | 0,22* | -0,02 |  |  |
| Physician | (13) | -0,19 | -0,10 | -0,05 | 0,00 | -0,19* | -0,31* | -0,20* | 0,15 | 0,04 | 0,00 | -0,09 | -0,39* |  |
| Supervisory position | (14) | -0,05 | -0,05 | 0,14 | 0,07 | 0,03 | 0,26* | 0,21* | 0,36* | 0,33* | 0,03 | -0,00 | -0,13 | 0,11 |
| *Note.* N=86; *p<0.05 |  |  |  |  |  |  |  |  |  |  |  |  |  |  |
