# Supplementary material for: The role of human capital and stress for cost awareness in the healthcare system: a survey among German hospital physicians
Source: BMC Health Serv Res. 2024 Mar 7;24:310. doi: 10.1186/s12913-024-10748-z (PMC10921634; doi:10.1186/s12913-024-10748-z)
Supplement: Supplementary file 2 — Supplementary Material 2 [file 12913_2024_10748_MOESM2_ESM.docx]

**Supplement**

**Online Survey**

1. **Estimation Questions**
2. **Please estimate the price (= revenue) of the following interventions:**
   1. Installation of a dual-chamber pacemaker
   2. 1 Colonoscopy
   3. 1 Coronary stenting
   4. 1 Cholecystectomy
3. **Level of Economic Knowledge**
4. **Economic/health economics content was part of my education/studies**


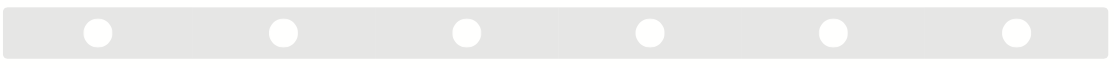


1

Strongly disagree

6

Strongly agree

1. **I have independently pursued further education in economic/health economics content**


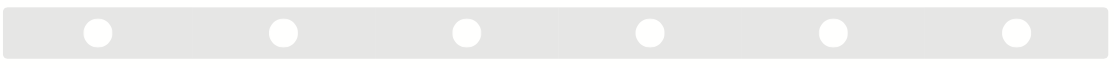


1

Strongly disagree

6

Strongly agree

1. **Please rate your level of knowledge of economic topics**
   1. Controlling/Accounting
   2. Marketing/Communication
   3. Insurance
   4. Taxes
   5. Human Resources

*1= Not existent to 6=Very well*

1. **I think economically when using material**


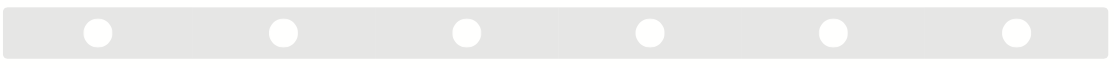


1

Strongly disagree

6

Strongly agree

1. **What is your total work experience? (In full years)**
2. **Stress**

**It happens that...**

1. **... I cannot respond to the patients' wishes or problems due to time constraints.**


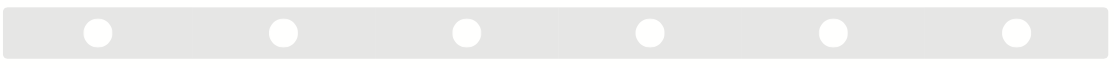


1

Strongly disagree

6

Strongly agree

1. **.. I am completely physically exhausted after work**


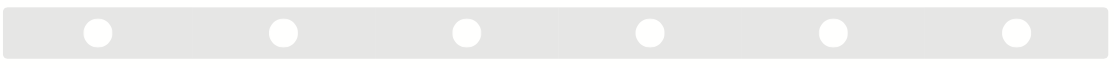


1

Strongly disagree

6

Strongly agree

1. **... I feel I have to take on too much responsibility**


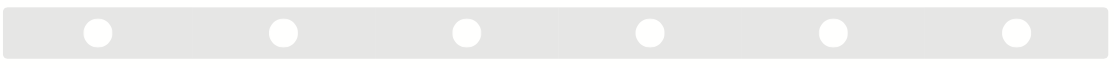


1

Strongly disagree

6

Strongly agree

1. **... I have the feeling that the work is so much that I can not cope with it**


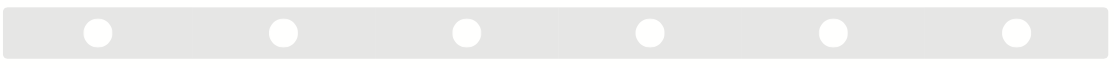


1

Strongly disagree

6

Strongly agree

1. **... I have to comply with a regulation that, in my opinion, does not make sense**


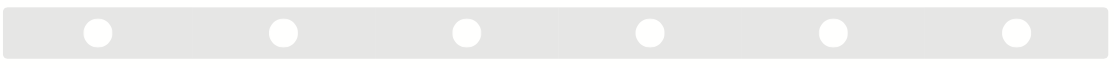


1

Strongly disagree

6

Strongly agree

**Cost-related stress**

1. **I can pursue my work free of economic pressure**


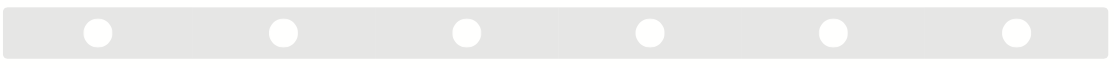


1

Strongly disagree

6

Strongly agree

1. **Demographic data**
2. **What is your gender?**

Male

Female

1. **How old are you?**
2. **What is your job title?**

Support staff (e.g. MFA, physician assistant, etc.)

Health care and nursing staff without specialization

Health care and specialist nurse

Assistant physician

Medical specialist

Senior physician

1. **Please name your field of expertise:**

Internal Medicine/Cardiology

Anesthesia

Orthopedics/Casualty Surgery

General surgery/Visceral surgery

Neurology

Urology

Gynecology

ENT/Ophthalmology

Other

1. **Are you in a supervisory position?**
   1. Yes
   2. No 
