# Supplementary material for: The role of human capital and stress for cost awareness in the healthcare system: a survey among German hospital physicians
Source: BMC Health Serv Res. 2024 Mar 7;24:310. doi: 10.1186/s12913-024-10748-z (PMC10921634; doi:10.1186/s12913-024-10748-z)
Supplement: Supplementary file 3 — Supplementary Material 3 [file 12913_2024_10748_MOESM3_ESM.docx]

**Appendix**

| **Table A1. Results of Robustness Tests: Probit Regression and Rare Event Logistic Regression with Price Estimation as Dependent Variable** | | | | |
| --- | --- | --- | --- | --- |
|  | **Probit Regression** |  | **Rare Events Logistic Regression** |  |
| General human capital | -0,05 |  | -0,10 |  |
|  | (0,05) |  | (0,09) |  |
| Job-Specific human capital | 0,00 |  | 0,00 |  |
|  | (0,00) |  | (0,01) |  |
| Domain-specific human capital | **-0,30** | ****** | **-0,62** | ******* |
|  | **(0,12)** |  | **(0,20)** |  |
| Domain-specific human capital squared | **0,06** | ******* | **0,13** | ******* |
|  | **(0,02)** |  | **(0,04)** |  |
| Own training in economics | -0,03 |  | -0,06 |  |
|  | (0,05) |  | (0,09) |  |
| Thinking economically when using material | **0,06** | ***** | **0,11** | ***** |
|  | **(0,03)** |  | **(0,05)** |  |
| Stress | **-0,13** | ******* | **-0,22** | ******* |
|  | **(0,04)** |  | **(0,07)** |  |
| Cost-related stress | 0,01 |  | 0,03 |  |
|  | (0,06) |  | (0,11) |  |
| Gender | -0,25 |  | -0,44 |  |
|  | (0,19) |  | (0,36) |  |
| Physician | -0,07 |  | -0,12 |  |
|  | (0,21) |  | (0,40) |  |
| Supervisory position | -0,20 |  | -0,33 |  |
|  | (0,18) |  | (0,34) |  |
| constant | -0,14 |  | 0,13 |  |
|  | (0,30) |  | (0,53) |  |
| Note: **p*>0.10, ***p*>0.05; ****p*>0.01; analyzed with 344 ratings for each regression model | | | | |
